# Better off breathing: an explanation for the seemingly detrimental impact of aerobic respiration on *Zymomonas mobilis*

**DOI:** 10.1128/mbio.02690-23

**Published:** 2023-12-20

**Authors:** James B. McKinlay

**Affiliations:** 1 Department of Biology, Indiana University, Bloomington, Indiana, USA; California Institute of Technology, Pasadena, California, USA

**Keywords:** *Zymomonas*, respiration, fermentation, oxygen toxicity, bacterial physiology, ethanol, polyploidy

## Abstract

The bacterium *Zymomonas mobilis* is best known for fermentatively producing more ethanol than yeast. However, *Z. mobilis* has also puzzled researchers for decades with the counterintuitive observation that disrupting aerobic respiration benefits aerobic growth, implying that fermentation remains favorable. Retention of detrimental respiration genes seemed to defy natural selection. New findings by Felczak et al. help clarify the importance of respiration for *Z. mobilis* and the factors that led to the confusing prior results (M. M. Felczak, M. P. Bernard, and M. A. TerAvest, 2023, mBio 14:e02043-23, https://doi.org/10.1128/mbio.02043-23). The team overcame redundancy from multiple genome copies to delete what turned out to be a key terminal oxidase. Unlike previous studies, wherein mutants exhibited low respiration rates and had improved aerobic growth, this mutant was incapable of respiration and had poor aerobic growth. Thus, respiration is important but surprisingly exceeds what is optimal under lab conditions. Respiration likely protects against toxic effects of oxygen, ensuring retention of respiration genes in the *Z. mobilis* genome.

## COMMENTARY

The bacterium *Zymomonas mobilis* is best known for its natural ability to ferment glucose into ethanol at 97% of the theoretical maximum yield. If not for this highly practical feature, *Z. mobilis* might have been better known as a model bacterium for mind-boggling traits, many of which have been summarized by Kalnenieks (1). Perhaps most confounding is that disrupting aerobic respiration, either via mutation or with cyanide, improves *Z. mobilis* growth under aerobic conditions (2–5). Such a counterintuitive observation forces one to question how respiration genes have been retained in the *Z. mobilis* genome. Moreover, if more reasons were needed to rid a genome of respiration genes, consider that in *Z. mobilis*, (i) respiration competes with fermentation under aerobic conditions, which can lead to accumulation of toxic acetaldehyde (1, 3); (ii) genes for aerobic respiration are still expressed under anaerobic conditions (6, 7); and (iii) respiration makes only a subtle contribution to cellular energy, to the extent that ATP levels can be higher in the presence of cyanide (1, 2). Taken together, decades of research seemed to suggest that *Z. mobilis* is better off without breathing.

How can constitutively expressed, detrimental respiration genes be maintained in the *Z. mobilis* genome? Unless we grant *Z. mobilis* a pass on natural selection, respiration must be important for *Z. mobilis* but for reasons that have long escaped us. Some insights have emerged in recent years. Work by the Rutkis et al. found that aerobic respiration increases the already notorious metabolic speed of *Z. mobilis* (8), which could impart a competitive advantage for resource control in nature. The same study found that aerobic respiration enhanced survival in non-growing conditions, suggesting that the selective advantage could be through avoiding death (8). More recently, my group found that posessing NADH dehydrogenase, encoded by *ndh* and the enzyme that supplies most of the electrons for aerobic respiration (5), was only disadvantageous for aerobic growth of *Z. mobilis* in a rich undefined medium (e.g., containing yeast extract) (3). However, *ndh* was critical for survival in a defined minimal medium (3). Even wild-type *Z. mobilis* grew poorly in the aerobic minimal medium, leading us to speculate that aerobic respiration is important in environments that lack compounds that can protect *Z. mobilis* against an unknown toxic effect of oxygen (O_2_). Thus, aerobic respiration might be detrimental in the lab but essential in natural environments.

While the above observations remain important insights, Felczak et al. have now exposed an experimental caveat that unknowingly influenced the interpretation of prior results where disrupting respiration led to improved growth (9). In previous work, whether cyanide was used or respiration genes were mutated, respiration was severely impaired but not eliminated. For example, genetic disruption of *ndh* drops the respiration rate to ~10% of that of wild type (5), a value that Felczak et al. verified. Surely such a drastic reduction of aerobic activity would be sufficient to inform on a role for aerobic respiration. Surely 10% of the wild-type respiration rate would be far from the optimum. What seemed like reasonable assumptions have now been defied by Felczak et al., who brought the respiration rate to zero.

Eliminating respiration was not straightforward. Although *Z. mobilis* genetics have improved in recent years, there are multiple genome copies per cell (polyploidy) to contend with, making it difficult to achieve true gene deletions (10). This is especially true for genes that are conditionally essential. Indeed, Felczak et al. easily deleted *ndh*, but deleting *cydAB*, encoding a terminal oxidase in the respiratory chain, proved stubborn. With perseverance and careful scrutiny to ensure that every copy of *cydAB* was eliminated, Felczak et al. obtained a bonafide *cydAB* mutant that, unlike previously reported mutants, was essentially incapable of any O_2_ reduction. Unlike the low-respiring *ndh* mutant that showed improved aerobic growth, the non-respiring *cydAB* mutation showed poor aerobic growth. Moreover, these trends were observed in a rich undefined medium, indicating that the yeast extract compounds that protected an *ndh* mutant (3) were insufficient to protect a *cydAB* mutant. Thus, respiration is critical for aerobic growth even in rich undefined media. But, consistent with the confusing fashion of *Z. mobilis*, the low respiration rate of the *ndh* mutant is potentially closer to the optimum than the wild-type respiration rate.

With the record corrected and aerobic respiration now identified as important for aerobic growth, Felczak et al. made headway on addressing what critical role aerobic respiration plays. Two non-exclusive hypotheses are that respiration contributes to cell energetics and/or protection against a toxic aspect of O_2_. As noted above, *Z. mobilis* respiration makes only a minor contribution to energy metabolism, but it nonetheless still contributes to the proton motive force, (1) which Felczak et al. verified. To separate O_2_ reduction from energy transformation, Felczak et al. expressed a heterologous cytoplasmic oxidase, encoded by *noxE*. NoxE couples NADH oxidation to O_2_ reduction but it is disconnected from any proton-translocating enzymes. Expressing the *noxE* gene improved the aerobic growth of a *cydAB* mutant, though not to wild-type levels. Thus, O_2_ removal alone is important for aerobic growth by *Z. mobilis*, but additional contributions of respiration are likely also important. The importance of O_2_ removal appeared to be separate from avoiding acetaldehyde toxicity because acetaldehyde was not detected for any of the respiration mutants used by Felczak et al. Reactive oxygen species were also not elevated in any of the respiration mutants. Thus, O_2_ itself is most likely a growth-inhibiting factor for *Z. mobilis*. Felczak et al. speculate that a critical O_2_-sensitive enzyme is behind the poor growth phenotype; O_2_-sensitive enzymes have been identified in other bacteria that are often thought of as anaerobes (11).

What enzyme or enzymes are critically sensitive to O_2_ in *Z. mobilis*? That question remains open for now, as do several others. For example, does the ability of respiration to protect *Z. mobilis* from O_2_ depend on a threshold cell density, as has been predicted elsewhere (11)? A threshold density could explain why both respiration and cell aggregation were important for survival of a *Z. mobilis ndh* mutant in a minimal medium (3). It is also curious why the wild-type respiration rate exceeded what seemed beneficial. The trend could be dependent on conditions, as the respiration rate without *ndh* was suboptimal for survival under non-growing conditions, particularly in the absence of protective factors in yeast extract (3, 8). A clear contribution of respiration to cell energetics also remains enigmatic for now. An important role for respiration in energy transformation could help explain why NoxE activity could not fully restore aerobic growth trends, despite imparting higher respiration rates than those by an *ndh* mutant, which seemed closer to optimal. With Felczak et al. having now solidified the importance of respiration for *Z. mobilis* and with trustworthy respiration mutants created, the field is now better equipped to make sense of the remaining unusual physiological traits possessed by this highly practical bacterium.
